# Prevalence, risk factors, and short-term outcome of contrast-induced nephropathy among patients with chronic kidney disease: A cross-sectional study from Sri Lanka

**DOI:** 10.1097/MD.0000000000043892

**Published:** 2025-08-15

**Authors:** Nalaka Herath, Premil Rajakrishna, L. Suriyaarachi, Sujani Premathilaka, Nishamadhu Perera, Sameera Samarasinghe, Shamila De Silva, Kosala Weerakoon

**Affiliations:** a Nephrology Unit, Teaching Hospital, Kurunegala, Sri Lanka; b Nephrology Unit, Colombo North Teaching Hospital, Ragama, Sri Lanka; c Department of Medicine, Faculty of Medicine, University of Kelaniya, Ragama, Sri Lanka; d Department of Parasitology, Faculty of Medicine and Allied Sciences, Rajarata University of Sri Lanka, Saliyapura, Sri Lanka.

**Keywords:** chronic kidney disease, CKD, contrast-induced nephropathy, Sri Lanka

## Abstract

Contrast-induced nephropathy (CIN) is a significant complication following the administration of radiocontrast media, defined as acute deterioration of renal function within 24 to 48 hours post-procedure. It represents the third most common cause of hospital-acquired acute kidney injury, accounting for approximately 11% of cases. CIN increases short-term morbidity and mortality while accelerating the progression of chronic kidney disease (CKD). This study investigated the prevalence, risk factors, and short-term outcomes of CIN among CKD patients undergoing contrast studies at 2 tertiary care centers in Sri Lanka. This descriptive cross-sectional study was conducted at the nephrology units of Colombo North Teaching Hospital and Teaching Hospital Kurunegala from August 2018 to September 2023. The study included 405 CKD patients over 18 years referred for pre-procedural nephrology consultation before undergoing contrast-enhanced imaging. All patients received intravenous normal saline hydration (1 mL/kg/h) 12 hours before and after contrast administration, with temporary discontinuation of nephrotoxic medications. Data was collected via an interviewer-administered questionnaire. CIN was defined per KDIGO guidelines as a rise in serum creatinine >25% or 0.5 mg/dL from baseline. The mean age of patients was 62.6 years (SD = 12.4), with 310 (76.5%) being male. Comorbidities included diabetes mellitus (48.9%), hypertension (44.2%), coronary artery disease (13.3%), and chronic liver disease (6.2%). CIN occurred in 45 patients (11.1%), with diabetes mellitus identified as a significant risk factor (*P* = .0044). Arterial contrast administration had a higher risk (17.7%) than venous administration (10.7%). Seven patients (1.7%) required acute hemodialysis, and 1 patient died. Age, gender, and CKD stage did not significantly influence CIN development. CIN remains a common complication in CKD patients undergoing contrast studies, with diabetes mellitus being a significant independent risk factor. Pre-procedural risk assessment and appropriate prophylactic measures are essential to minimize CIN incidence and associated complications.

## 1. Introduction

Radiological investigations involving contrast media play a crucial role in both diagnostic and therapeutic procedures in modern healthcare. While contrast medium enhances imaging accuracy, its administration is associated with a significant complication known as contrast-induced nephropathy (CIN).^[1]^ CIN is defined by the Kidney Disease Improving Global Outcomes (KDIGO) guidelines as an acute deterioration of renal function, characterized by a rise in serum creatinine >25% or 0.5 mg/dL (44 μmol/L) from baseline within 24 to 48 hours following contrast administration.^[2]^

CIN is the third most prevalent cause of hospital-acquired acute kidney injury, accounting for approximately 11% of cases.^[3]^ Its clinical significance extends beyond the acute phase, as it increases both short-term morbidity and mortality and potentially accelerates the progression of preexisting chronic kidney disease (CKD) over the long term.^[3,4]^ This dual impact makes prevention and early identification of CIN particularly important in clinical practice.

Previous studies have identified multiple risk factors for developing CIN, including preexisting renal disease, diabetes mellitus, hypertension, congestive heart failure, liver cirrhosis, and advanced age.^[5,6]^ Contrast-related factors such as the type of contrast agent, route of administration, and dose also influence the likelihood of developing CIN.^[7,8]^ Understanding these risk factors is crucial for implementing appropriate preventive strategies.

Current preventive approaches primarily focus on patient hydration and using low-osmolar or iso-osmolar contrast media.^[2]^ Despite these measures, CIN remains a significant concern in clinical practice due to its association with adverse outcomes, including prolonged hospital stays and increased healthcare costs.^[1]^

Sri Lanka faces a unique burden of CKD, including CKD of unknown etiology, making the study of contrast-related complications particularly relevant. The prevalence of CKD of unknown etiology in the North Central Province of Sri Lanka, the worst affected area of the country, is between 15.1% and 22.9%, with the highest prevalence among those aged 40 years and above.^[9]^ Epidemiological data on chronic kidney disease in the country is lacking; however, the Ministry of Health estimates that approximately 10% of the population in Sri Lanka is afflicted by kidney-related ailments.^[10]^ Given this high burden of renal disease in Sri Lanka, especially in endemic areas, the risk of CIN is a significant concern during radiological procedures. Recognizing CIN is pivotal in preventing iatrogenic harm among vulnerable patients, in these settings.

While CIN has been extensively studied in developed countries, there is limited data from South Asian populations, particularly Sri Lanka. This research gap necessitates specific investigation into CIN prevalence and risk factors in the Sri Lankan healthcare context. Therefore, this study aimed to investigate the prevalence, risk factors, and short-term outcomes of CIN among patients with CKD undergoing iodine-based contrast studies with pre- and post-intravenous hydration with normal saline at 2 major tertiary referral centers in Sri Lanka.

## 2. Methods

This descriptive cross-sectional study was conducted at the nephrology units of Colombo North Teaching Hospital, Ragama and Teaching Hospital, Kurunegala from August 2018 to September 2023. The 2 units function as key tertiary care referral centers in the Western and North-western provinces of Sri Lanka, respectively.

All consecutive adult patients (≥18 years) diagnosed with CKD stages 2 to 5 referred for pre-procedural nephrology consultation before undergoing contrast-enhanced imaging and able to provide informed written consent were considered for inclusion throughout the study period, based on defined inclusion and exclusion criteria. Patients with prior exposure to contrast media within the preceding 2 weeks, those with acute kidney injury at baseline, and pregnant patients were excluded.

Information was collected using an interviewer-administered questionnaire after obtaining informed written consent. The data gathered included demographic information (age, gender), medical history and comorbidities, CKD stage based on estimated glomerular filtration rate (eGFR), type of contrast study performed, renal function parameters before and 24 and 48 hours after contrast administration, and clinical outcomes including the need for kidney replacement therapy.

By KDIGO guidelines for preventing CIN, strict protocols were followed for all patients.^[11]^ Intravenous normal saline hydration was administered 12 hours before and after the contrast procedure, with the rate adjusted to 1 mL/kg/h based on the patient’s body weight. Nonsteroidal anti-inflammatory drugs, ACE inhibitors, angiotensin receptor blockers and metformin were temporarily discontinued at least 24 hours before the contrast study. Low-osmolar or iso-osmolar contrast media was used in all procedures. Contrast volume was minimized according to procedure requirements. Serum creatinine was measured at baseline, 24 and 48 hours post-procedure. CIN was defined according to KDIGO guidelines as a rise in serum creatinine >25% or 0.5 mg/dL (44 μmol/L) from baseline within 48 hours following contrast administration.^[2]^

The ethical review committee of the Faculty of Medicine, University of Kelaniya granted ethical clearance (P/216/12/2018) for this study, as did the ethical review committee of Teaching Hospital, Kurunegala (THK/HIRU/ERC/2021/02). All participants provided informed written consent before enrollment.

Data analysis was performed after all collected information was entered into a Microsoft Excel database. Continuous variables were presented as means with standard deviations, while categorical variables were presented as frequencies and percentages. Comparisons between groups were performed using Student *t* test for continuous variables and chi-square test for categorical variables. Odds ratios (OR) with 95% confidence intervals (CI) were calculated to assess the strength of associations. A *P*-value <.05 was considered statistically significant. Multivariate logistic regression analysis was performed to identify independent risk factors for CIN development after adjusting for potential confounders.

## 3. Results

### 3.1. Demographic and clinical characteristics

During the study period, 405 patients with CKD were referred to the nephrology services in both hospitals and were included in the analysis. The cohort’s mean age was 62.6 years (standard deviation = 12.4), and 310 patients (76.54%) were male.

The distribution of CKD stages was as follows:

- CKD stage 2 (eGFR 60–89 mL/min/1.73 m^2^): 41 patients (10.12%);
- CKD stage 3 (eGFR 30–59 mL/min/1.73 m^2^): 261 patients (64.44%);
- CKD stage 4 (eGFR 15–29 mL/min/1.73 m^2^): 83 patients (20.49%);
- CKD stage 5 (eGFR <15 mL/min/1.73 m^2^): 20 patients (4.94%).

The most common comorbidities included diabetes mellitus (198 patients, 48.9%), hypertension (179 patients, 44.2%), coronary artery disease (54 patients, 13.3%), and chronic liver cell disease (25 patients, 6.2%).

### 3.2. Contrast procedures

The diagnostic imaging modalities utilized in the study included:

- CT of abdomen and pelvis (122 patients, 30.1%);
- CT IVU (84 patients, 20.7%);
- CT chest (70 patients, 17.3%);
- Coronary angiogram (51 patients, 12.6%);
- CT head (44 patients, 10.9%);
- CT chest/abdomen/pelvis (34 patients, 8.4%).

### 3.3. Prevalence and risk factors for CIN

Of the 405 patients, 45 (11.1%) developed CIN according to KDIGO criteria. The mean age of patients who developed CIN was 63.8 years (range 30–93 years), and 30 (67%) were male.

Patients with diabetes mellitus had a significantly higher risk of developing CIN than those without diabetes (OR 2.56, 95% CI 1.32–4.97, *P* = .006). Age and gender did not significantly influence CIN development (Table 1).

**Table 1:** Comparison of characteristics between patients who did and did not develop CIN.

Patient characteristics	Total group (n = 405)	Developed CIN (n = 45)	Did not develop CIN (n = 360)	Statistical significance
Mean age in years (range)	62.55 (23–93)	63.84 (30–93)	62.44 (23–89)	*P* = .238
Male	310 (76.54%)	30 (66.67%)	280 (77.78%)	OR 0.57 (95% CI 0.29–1.11, *P* = .1)
Female	95 (23.46%)	15 (33.33%)	80 (22.22%)	
CKD stage				
Stage 2	41 (10.12%)	10 (22.22%)	31 (8.61%)	OR 3.03 (95% CI 1.37–6.70, *P* = .006)
Stage 3	261 (64.44%)	24 (53.33%)	237 (65.83%)	OR 0.65 (95% CI 0.35–1.21, *P* = .18)
Stage 4	83 (20.49%)	9 (20.0%)	74 (20.56%)	OR 0.97 (95% CI 0.45–2.10, *P* = .93)
Stage 5	20 (4.94%)	2 (4.44%)	18 (5.0%)	OR 0.88 (95% CI 0.19–3.94, *P* = .87)
Comorbidities				
Diabetes mellitus	198 (48.89%)	31 (68.89%)	167 (46.39%)	OR 2.56 (95% CI 1.32–4.97, *P* = .006)
Hypertension	179 (44.20%)	19 (42.23%)	160 (44.44%)	OR 0.91 (95% CI 0.49–1.71, *P* = .777)
Coronary artery disease	54 (13.33%)	7 (15.56%)	47 (13.06%)	OR 1.23 (95% CI 0.52–2.91, *P* = .642)
Chronic liver disease	25 (6.17%)	4 (8.89%)	21 (5.83%)	OR 1.58 (95% CI 0.52–4.78, *P* = .423)

CIN = contrast-induced nephropathy, CKD = chronic kidney disease.

The route of contrast administration was found to influence the risk of CIN. Arterial contrast administration (coronary angiogram) carried a higher risk (17.65%) compared to venous administration (10.65%) (Table 2).

**Table 2:** Development of CIN by route and type of contrast study.

Route	Contrast study	Developed CIN (n = 45)	Did not develop CIN (n = 360)	Risk of developing CIN
Venous study	CECT brain/head	6	44	13.64%
	CECT chest	8	70	11.43%
	CECT abdomen/pelvis	16	122	13.11%
	CECT chest/abdomen/pelvis	4	28	14.29%
	CECT IVU	2	84	2.38%
Total venous		36	348	10.65%
Arterial study	Coronary angiogram	9	51	17.65%
Total arterial		9	51	17.65%

CECT = contrast enhanced CT, CIN = contrast-induced nephropathy, IVU = intravenous urogram.

Multivariate logistic regression analysis confirmed diabetes mellitus as an independent risk factor for CIN development (adjusted OR 2.43, 95% CI 1.26–4.68, *P* = .008) after adjusting for age, gender, CKD stage, and route of contrast administration.

### 3.4. Clinical outcomes

Among the 45 patients who developed CIN, 7 (15.6% of CIN cases, 1.72% of the total cohort) required acute intermittent hemodialysis due to severe renal impairment. One patient (2.2% of CIN cases) died due to multiple comorbid conditions. The remaining 37 patients showed improvement in renal function without needing kidney replacement therapy.

At discharge, the 7 patients who required hemodialysis had not regained their previous level of renal function while 37 patients were at their baseline renal function. None of the patients required long-term dialysis.

## 4. Discussion

Our study revealed an 11.1% prevalence of CIN among CKD patients undergoing contrast studies at 2 tertiary care centers in Sri Lanka, comparable to previous studies’ findings.^[1]^ This prevalence highlights the importance of recognizing CIN as a significant complication in patients with preexisting renal impairment.

The findings of our study contribute valuable data to the limited literature on CIN in South Asian populations. The prevalence of CIN in our cohort (11.1%) aligns with global estimates, suggesting that despite regional variations in healthcare delivery and patient characteristics, the fundamental pathophysiology and risk factors for CIN may be consistent across populations.

Diabetes mellitus emerged as a significant independent risk factor for CIN in our study population, a finding consistent with previous research.^[4,5]^ Patients with diabetes have increased susceptibility to renal injury due to diabetic nephropathy, altered renal hemodynamics, and increased oxidative stress, all of which may potentiate contrast-induced renal damage.^[12]^ This emphasizes the importance of special precautions when administering contrast media to diabetic patients. Interestingly, while previous studies have suggested associations between CIN and factors such as advanced age, female gender, and more severe CKD,^[2]^ our study did not find significant correlations with these variables. This discrepancy may reflect the strict preventive protocols implemented in our study, which might have mitigated the influence of these traditional risk factors. A number of patients developed CIN with preserved GFR (eGFR > 60 mL/min/1.73 m^2^). This may be due to diabetic kidney disease and heavy proteinuria. However, there was inadequate information regarding this group of patients for better characterization of risk factors.

Our study also demonstrated a higher risk of CIN following arterial contrast administration (17.65%) than venous administration (10.65%). This finding aligns with the results reported by Chaudhury et al,^[1]^ who found that intra-arterial contrast poses a greater risk for renal injury compared to intravenous administration. The increased risk associated with arterial administration may be attributed to higher intrarenal contrast concentrations and direct endothelial damage.

All patients in our study received pre- and post-procedure hydration with intravenous normal saline according to KDIGO guidelines.^[11]^ This standardized approach likely contributed to the relatively favorable outcomes observed in our cohort. Prior to contrast studies, the systematic discontinuation of nephrotoxic and potentially nephrotoxic medications may have also played a protective role. While isotonic sodium bicarbonate represents an alternative hydration fluid with theoretical advantages in preventing CIN,^[11,13]^ our study utilized normal saline as it is more readily available and cost-effective in resource-limited settings like Sri Lanka.

This study has several limitations. The observational design precludes definitive conclusions about causality. The study was conducted at 2 tertiary care centers, potentially limiting generalizability to other healthcare settings in Sri Lanka. Long-term follow-up data beyond hospital discharge were not available, limiting our understanding of the persistent effects of CIN on CKD progression. Information about contrast volumes was not consistently documented for all procedures. Potential confounding factors such as concurrent medications and hemodynamic status during procedures were not comprehensively assessed. Information on baseline proteinuria, blood pressure, and use of RAAS blockers was also not available in this cohort.

Based on our findings, we recommend a thorough risk assessment before contrast administration in CKD patients, with particular attention given to diabetic status and the route of contrast administration. For high-risk patients, consideration should be given to alternative imaging modalities that do not require iodinated contrast.

Future research should focus on long-term outcomes of patients who develop CIN, particularly regarding CKD progression, comparative effectiveness of different preventive strategies in the Sri Lankan population, development of risk prediction models specific to South Asian populations, investigation of novel biomarkers for early detection of CIN and assessment of the cost-effectiveness of preventive strategies in resource-limited settings.

## 5. Conclusions

Contrast-induced nephropathy is a common complication in patients with chronic kidney disease who undergo iodine-based contrast studies, with an incidence of 11.1% in this Sri Lankan cohort. Diabetes mellitus represents a significant independent risk factor for CIN, and arterial contrast administration carries a higher risk compared to venous administration. Most patients with CIN recover without requiring kidney replacement therapy. These findings emphasize the importance of comprehensive risk assessment before performing contrast studies in CKD patients and considering alternatives in high-risk patients.

## Acknowledgments

The authors are most grateful to Dr Rajeewa Dassanayake, Dr Laxman Senarathne, Dr Asanka Kumara, Dr Tharanga Dilhani, Dr Dilusha Nilmini, Dr Erandi Warnasooriya, Dr Kasun Sooriyarachchi, Dr Sudhammi Perera, Dr Prasitha Liyanage, Dr Sameera Kumara, Dr Suganthika Devi, Dr Ruwani Mallawarachi, Dr Iresha Karunathilaka, and Miss Jayani Lankathilaka, for their help in carrying out this research.

## Author contributions

**Conceptualization:** Nalaka Herath.

**Data curation:** Premil Rajakrishna, L. Suriyaarachi, Sujani Premathilaka, Nishamadhu Perera, Sameera Samarasinghe.

**Formal analysis:** Nalaka Herath, Shamila De Silva, Kosala Weerakoon.

**Investigation:** Nalaka Herath, Premil Rajakrishna, L. Suriyaarachi, Sujani Premathilaka, Nishamadhu Perera, Sameera Samarasinghe, Shamila De Silva, Kosala Weerakoon.

**Methodology:** Nalaka Herath, Shamila De Silva, Kosala Weerakoon.

**Project administration:** Nalaka Herath.

**Supervision:** Nalaka Herath.

**Validation:** Nalaka Herath, Shamila De Silva, Kosala Weerakoon.

**Writing – original draft:** Nalaka Herath, Shamila De Silva, Kosala Weerakoon.

**Writing – review & editing:** Nalaka Herath, Shamila De Silva, Kosala Weerakoon.

## References

[1] Chaudhury P Armanyous S Harb SC. Intra-arterial versus intravenous contrast and renal injury in chronic kidney disease: a propensity-matched analysis. Nephron J. 2018;141:31–40. 10.1159/000494047 30368506

[2] KDIGO clinical practice guideline for acute kidney injury. 2012;2. http://www.kidney-international.org. Accessed January 30, 2025.

[3] Nash K Hafeez A Hou S. Hospital-acquired renal insufficiency. Am J Kidney Dis. 2002;39:930–6. 11979336 10.1053/ajkd.2002.32766

[4] McCullough P. Outcomes of contrast-induced nephropathy: experience in patients undergoing cardiovascular intervention. 2006. https://onlinelibrary.wiley.com/doi/abs/10.1002/ccd.20658. Accessed January 30, 2025. 10.1002/ccd.20658 16489569

[5] Rudnick MR Goldfarb S Tumlin J. Contrast-induced nephropathy: is the picture any clearer? Clin J Am Soc Nephrol. 2008;3:261–2. 18077780 10.2215/CJN.04951107

[6] Herath N Liyanage P Kumara S. Risk factors for contrast nephropathy and outcome at a tertiary referral centre in Sri Lanka. Kidney Int Reports. 2020;5:S20.

[7] Polena S Yang S Alam R. Nephropathy in critically ill patients without preexisting renal disease. Proc West Pharmacol Soc. 2005;48:134–5. 16416679

[8] Cigarroa RG Lange RA Williams RH Hillis LD. Dosing of contrast material to prevent contrast nephropathy in patients with renal disease. Am J Med. 1989;86:649–52. 2729314 10.1016/0002-9343(89)90437-3

[9] Rao IR Bangera A Nagaraju SP. Chronic kidney disease of unknown aetiology: a comprehensive review of a global public health problem. Trop Med Int Health. 2023;28:588–600. 37403003 10.1111/tmi.13913

[10] Annual Health Bulletin 2022-2023. Sri Lanka medical statistics unit ministry of health. 2024. https://www.health.gov.lk/annual-health-bulletin/. Accessed January 30, 2025.

[11] Section 4: contrast-induced AKI. Kidney Int Suppl. 2012;2:69–88. 10.1038/kisup.2011.34 PMC4089629 25018920

[12] Calvin AD Misra S Pflueger A. Contrast-induced acute kidney injury and diabetic nephropathy. Nat Rev Nephrol. 2010;6:679–88. 20877303 10.1038/nrneph.2010.116 PMC4476402

[13] Pannu N Wiebe N Tonelli M, Alberta Kidney Disease Network FT. Prophylaxis strategies for contrast-induced nephropathy. JAMA. 2006;295:2765–79. 16788132 10.1001/jama.295.23.2765

